# The Effect of Education Based on Health Belief Model on Health Beliefs of Women with Urinary Tract Infection

**Published:** 2014-01

**Authors:** Fereshteh Javaheri Tehrani, Soqra Nikpour, Eftekhar Alsadat Haji Kazemi, Neda Sanaie, Shabnam Alsadat Shariat Panahi

**Affiliations:** 1 Department of Community Health Nursing, Tehran University of Medical Sciences, Tehran, Iran;; 2 Department of Critical Care Nursing, Zanjan University of Medical Sciences, Zanjan, Iran

**Keywords:** Education, Health Belief Model, Women, Urinary Tract Infection

## Abstract

**Background: **Urinary Tract Infection is one of the commonest infections which affect humans. Half of all women have a UTI in their lifetime and one fourth have recurrent infections. Health behaviours can help patients to prevent Urinary Tract Infection recurrence and changing beliefs is necessary for health behaviour change. The aim of this study is to investigate the effect of education based on Health Belief Model on health beliefs of women with Urinary Tract Infection.

**Methods: **This is a quasi-experimental study with pre-test and post-test design, conducted on 170 married women with Urinary Tract Infection, referred to selected hospital laboratories in Tehran. The laboratories were divided to experience and control groups. The data collection tool was a “self-administrated” questionnaire which was answered by samples of both groups, prior to the intervention and 12 weeks thereafter. The intervention (education based on Health Belief Model) was performed on the experiment group.

**Results: **Based on the study results, after the intervention the average score of the perceived susceptibility (P<0.001), perceived severity (P<0.001), perceived benefits (P<0.001), cues to action (P<0.001) and health behaviours (P<0.001) of the experiment group showed a significant increase, compared to the control group, however, the average score of the perceived barriers (P=0.235) of the experiment group was not significantly different compared to the control group.

**Conclusion: **The findings showed that education based on Health Belief Model was effective in promoting the health beliefs (except perceived barriers) and health behaviours of women with Urinary Tract Infection. Therefore, it can be suggested that the mentioned model can be used as one of the strategies for prevention of Urinary Tract Infection in women.

## Introduction

Urinary Tract Infection (UTI) is one of the commonest infections which affect humans, cause significant distress to the individuals, and is associated with high healthcare and social costs.^1^ If UTI is not treated by primary health care providers, it can cause serious potential consequences, such as pyelonephritis (inflammation of the kidney) and bacteraemia (bacteria in the blood). In addition, it is believed that UTIs have become increasingly resistant to first-line antibiotic therapy.^2^

Between the ages 16 and 35, women are about 40 times more likely to develop a UTI than aged-matched males.^3^ A study in Canada showed that about one half of women have a UTI in their lifetime and one fourth experience recurrent infections.^4^

UTI is limited to the lower urinary tract system in most women^5^ which is known as Uncomplicated Urinary Tract Infections that are most common in patients with no structural or functional disorder of the urinary tract and kidneys,^6^ and occurs in patients who have a normal, unobstructed genitourinary tract, with no history of recent instrumentation^7^ and healthy premenopausal, non-pregnant women.^8^

It is said that Escherichia coli accounted for the great majority of infections.^9^ UTI is mainly caused by focal bacteria, 85% of which are E. coli. Escherichia coli are the most common cause of uncomplicated UTIs and accounts for approximately 75 to 95 percent of all infections.^7^

On the other hand, Escherichia coli are not only the first cause of UTIs in women, but they also increase the likelihood of recurrent UTI. Even after resolution of a UTI, small numbers of the original strain of uropathogens may persist in the host, allowing re-colonization and re-infection to occur.^10^ Therefore early diagnosis and immediate treatment of urinary tract infection is essential to prevent infection recurrence and its complications, including renal failure, sepsis, adhesions and obstruction and the purpose of the treatment is to prevent progression of infection and permanent damage and kidney failure.^11^

Since, sexual behaviours and health habits play an important role in causing UTI^12^ and predominant risk factors for recurrent UTIs in premenopausal women are behavioural risk factors.^10^ We can conclude that by educating UTI risk factors and changing health behaviours, we can take an important step in preventing UTI.^12^

On the other hand, people need assistance and training for changing their health behaviours,^13^ and the aim of health education according to Simonds (1976) is bringing about changes in behavioural patterns from detrimental to optimal, to behaviours that are conducive to present and future health; for health education to be effective, it should be designed with understanding of the recipient’s health and social characteristics, beliefs, attitudes, values, skills, and past behaviours.^14^

Beliefs and attitudes provided a link between socialization and behaviour. Beliefs are enduring individual characteristics which shape behaviour and are also modifiable. Therefore, if persuasive methods can be used to change behaviour-related beliefs, these interventions also result in behaviour change.^15^

Health theories and models have been valued in the field of health promotion and are important in explaining health risk factors and changing behaviour and its methods.^16^ All forms of public health intervention are not equally successful in achieving their aims and objectives, but other studies showed that the use of theory significantly improves the chances for success in achieving objectives.^15^

The HBM has been used extensively to determine the relationships between health beliefs and health behaviours. For example, some studies have been significantly associated with greater perceived susceptibility, lower barriers, higher benefits and cues in the form of recommendations from health care providers.^14^

HBM focused on threat perception and behavioural evaluation. Actually the treat perception consists of two constructions; perceived susceptibility to health problem and perceived severity of the complications of diseases or health problems. Behavioural evaluation also consisted of two distinct sets of beliefs, those concerning the benefits of recommended health behaviour and those concerning the barriers to enact the behaviour. In addition, the model proposed that cues to action can activate health behaviour when appropriate beliefs are held.^15^ In fact, this model predicts that individuals will take action to protect or promote health if they perceive themselves to be susceptible to a condition or problem and it will have potentially serious consequences. Also when they believe a course of action is available which will reduce their susceptibility, the benefits of taking any action will outweigh the costs or barriers.^17^

The literature supports the importance of health education based on HBM on promotion of women’s beliefs regarding Breast Cancer prevention,^18^^-^^20^ Cervical Cancer,^21^ Gastric Cancer,^22^ diabetic patients^23^ and nulliparous women.^24^ In another study, education based on HBM could promote the attitudes of pregnant women regarding UTI as the first level prevention.^25^ But in our country less attention has been paid to the effect of education based on HBM on health beliefs of non-pregnant women with Urinary Tract Infection, particularly as a second level prevention. Therefore, this study was conducted to determine the effect of education based on Health Belief Model on health beliefs of women with Urinary Tract Infection, as the second level prevention.

## Materials and Methods

This study was approved by Medical Ethics Centre of Tehran University of Medical Sciences. This is a quasi-experimental study which has been conducted on 204 women with UTI who had referred to 4 selected hospital laboratories (Arash, Valiasr, Shariati, and Akbarabadi) in Tehran. The study was conducted over a period of six months in 2012. A total of 280 participants were enrolled in the study-148 subjects for the experiment group and 132 for the control group. Only 204 participants comprising of 102 samples in each group met the inclusion criteria and completed the questionaires. It should be mentioned that the sample size was estimated according to a previous study^26^ including a 20 percent drop in samples, 80 percent test power, 3 mean differences and 7 standard deviation as 204 samples (102 samples in each groups).

According to a study pattern,^27^ women, who had positive urine culture (colony counts more than 100,000), in less than three months prior to the launching of the study, were contacted by phone and invited to participate in the study. Women who were married, aged 20-45, non-pregnant, educated (ability to reading and writing), non-diabetics, without hypertension, polycystic kidney, kidney stones or any obstruction of urinary tract (diagnosed by physician), kidney transplant, and urinary incontinence, pre-menopause and with no history of hospitalization over the last three months, participated in the study. The others were excluded and received an educational booklet about UTI with regard to ethics.

Two hospitals with gynecology department and two Specialty hospitals selected in Tehran, and their laboratories were divided into two groups (experiment and control group). The intervention was held for the experiment group samples referred to Arash and Valiasr Hospital laboratories and no intervention was performed for the control group samples, referred to Shariati and Akbarabadi Hospital laboratories. Consent forms were taken before filling in the questionnaires. The samples in both groups filled in the questionnaires before the intervention and 12 weeks thereafter. Regarding ethics, the educational booklet about UTI was given to the control group at the end of the study.  

The data collection tool in this study was a self-administrated questionnaire, adapted from previous similar studies^25^^,^^28^ and library resources. Internal correlation of the questionnaire was calculated by a Statistical Software (SPSS 16) and reliability analysis test for different sections. Cronbach’s Alfa was 0.95, 0.86, 0.93 and 0.96 respectively for attitudes based on HBM (perceived susceptibility, perceived severity, perceived barriers and perceived benefits), cues to action, health behaviours and whole of the questionnaire. Then, its validity was examined by Tehran Nursing and Midwifery Faculty’s members (10 persons) and the questionnaire was revised again. Its reliability was estimated through test re-test and a pilot study. In the pilot study, 10 women who had experienced UTI previously filled in the questionnaire twice within ten days, and then we calculated Pearson correlation coefficient with 95% confidence interval as r=0.81. The pilot study was also used for educational needs assessment.

The questionnaire consisted of four parts. The first part was about demographic information and disease-related variables. The second part included questions on beliefs evaluation based on HBM which were about the perceived susceptibility, perceived severity, perceived barriers, and perceived benefits with the five-part Likert type scale as strongly agree (5 points), agree (4 points), no opinion (3 points), disagree (2 points) and strongly disagree (1 point). The third part was the cues to action including history of UTI, internet, radio or TV, books, newspapers or magazines, educational booklets, family or friends, healthcare workers and personal education which were answered with yes (1 point) and no (0 point) response pattern; the last part of the was about the health behaviours evaluation including 24 health behaviours about treatment adherence, diet, self hygiene, cloths health, sexual health and urination health which were answered with always (3 points),, sometimes (2 points), often (1 point) and never (0 point) options. The response evaluation criteria were quantitative.

The intervention included educational sessions (one per week for a total of 2 sessions) was performed for the experiment group samples.  The sessions were held for the groups of 10 to 15 persons. The subject of these educational sessions was about UTI and its epidemiology, pathogen, predisposing factors, symptoms, diagnosis, treatment, follow up and preventive health behaviours. PowerPoint Software was used to show the images related to the urinary tract structure. They received educational booklet, for further investigation. Various activities to enhance the beliefs of participants based on HBM were performed. To enhance their perceived susceptibility about UTI, we expressed the high prevalence of UTI in women specially married ones. We explained the complications of recurrent UTI such as renal failure, sepsis, adhesions, and so on to increase their perceived severity about UTI. The role of health behaviours to prevent recurrence of UTI was described to increase the perceived benefits of UTI preventive behaviours and the participants were encouraged to be active in group discussions about barriers of UTI preventive behaviours to compare the benefits and barriers and overcome the barriers. It should be mentioned that Educational booklet about UTI and preventive behaviours was as a cue to action for the samples. 

The obtained data were analyzed using SPSS 16 Software. Independent *t* test and Chi-square tests were used for description indexes and frequency distribution tables. Based on specific objectives of the study, the results were compared between the two groups, before and after the intervention. Based on Kolmogorov-Smirnov test result, independent *t* test was used for the normally distributed data and Mann Whitney test was used for the data with abnormal distribution. The P<0.05 was considered as the significance level.

## Results

170 samples comprising of 85 in each group participated until the end of the study and filled in the questionnaires once again. 17 samples in each group left the study due to lack of interest to continue participation and we no longer had access to them. 

Based on Chi-square test results, there was no significant difference between the experiment and control group samples in terms of demographic and disease-related variables. The mean age of the experiment group samples was 35.71±6.61 while it was 33.69±6.85 in the control group (P=0.249); most of them were housewives (P=0.269). The majority of the samples had attained a high school diploma (P=0.331); the marriage age of the majority was 20 to 29 years old (P=1.0); they had labor twice (P=0.341), had been hospitalized twice (P=0.420), and did not have history of hospital acquired Urinary Tract Infection or UTI after hospitalization (P=0.186). They first experienced UTI at the age of 21 to 25 (P=0.802); most of them had history of 2 times UTI diagnosed by a clinician requiring antimicrobial treatment (P=0.614). The pathogen of their recent UTI was E Coli bacteria (74.1 % in the experiment and 72.9%% in the control group) (P=0.717) (table 1).

**Table 1:** Distribution of most important demographic and disease-related variables in the Samples of the experiment and control groups

**Variables** **N (%)**		**Experiment**	**Control**	**Statistical test**	**P value**
		**N (%)**	**N (%)**		
Age (years)	20-29	19 (22.4)	28 (32.9)	Chi square	0.249
	30-39	38 (44.7)	36 (42.4)		
	40-45	28 (32.9)	21 (24.7)		
Education	Elementary	13 (15.3)	5 (5.9)	Chi square	0.331
	Junior high school	11 (12.9)	16 (18.8)		
	High school	5 (5.9)	5 (5.9)		
	Diploma	37 (43.5)	40 (47.1)		
	University degree	19 (22.4)	19 (22.4)		
Job	Housewife	69 (81.2)	63 (74.1)	Chi square	0.269
	Employed	16 (18.8)	22 (25.9)		
Number of pregnancy	0	14 (16.5)	15 (17.6)	Chi square	0.210
	1	14 (16.5)	23 (27.1)		
	2	30 (35.3)	26 (30.6)		
	3	13 (15.3)	15 (17.6)		
	More than 4	14 (16.5)	6 (7.1)		
Hospital acquired UTI	Yes	15 (17.6)	9 (10.6)	Chi square	0.186
	No	70 (82.4)	76 (89.4)		
Age of the first UTI diagnosis	<15	5 (5.9)	7 (8.2)	Chi square	0.802
	16-20	18 (21.2)	23 (27.1)		
	21-25	29 (34.1)	25 (29.4)		
	26-30	26 (30.6)	22 (25.9)		
	31-45	7 (8.2)	8 (9.4)		
Number of UTI diagnosis	1	10 (11.8)	12 (14.1)	Chi square	0.614
	2	31 (36.5)	34 (40)		
	3	21 (24.7)	14 (16.5)		
	4 and more	23 (27.1)	25 (29.4)		
Recent UTI pathogens	Escherichia Coli	63 (74.1)	62 (72.9)	Chi square	0.717
	Staphylococcus aurous	2(2.4)	3 (3.5)		
	Staphylococcus saprophyticus	14 (16.5)	9 (10.6)		
	Staphylococcus epidermidis	2 (2.4)	3 (3.5)		
	Klebsiella pneumonia	3 (3.5)	5 (5.9)		
	Enterococcus faecalis	1 (1.2)	3 (3.5)		

Based on the results of the statistical tests (independent *t* test and Mann-Whitney test), it was found out that the scores of the perceived susceptibility (P=0.500), perceived severity (P=0.186), perceived barriers (P=0.597) perceived benefits (P=0.186), cues to action (P=0.823) and health behaviours (P=0.821) showed no significant difference between the experiment and control groups before the intervention. Although the scores of the perceived susceptibility (P<0.001), perceived severity (P<0.001), perceived benefits (P<0.001), cues to action (P<0.001) and health behaviours (P<0.001) had significantly increased after the intervention in the experiment group compared to the control group, the perceived barriers score of the experiment group had no significant difference compared to the control group after the intervention (P=0.235) (table 2).

**Table 2:** Control between the mean and standard deviation of HBM components and health behaviours in the experiment and control groups before and after the intervention

**Variables**	**Experiment**	**Control**	**Statistic** **al test** **(between two groups)**
Perceived susceptibility before the intervention	14.37±2.05	14.10±2.16	Mann Whitney Test P=0.500 Z=-0.675
Perceived susceptibility after the intervention	17.09±2.76	14.77±2.03	Mann Whitney Test P<0.001 Z=-6.075
Perceived severity before the intervention	16.28±2.01	15.51±3.08	Mann Whitney Test P=0.186 Z=-1.321
Perceived severity after the intervention	18.17±1.95	15.87±2.08	Mann Whitney Test P<0.001 Z=-6.863
Perceived barriers before the intervention	10.92±3.26	10.62±3.18	Independent *t* test P=0.597 t=0.619
Perceived barriers after the intervention	9.11±3.31	9.41±3.59	Independent *t* test P=0.235 t=-0.554
Perceived benefits before the intervention	13.55±1.70	12.89±2.50	Mann Whitney Test P=0.186 Z=-1.322
Perceived benefits after the intervention	14.38±1.26	13.36±1.64	Mann Whitney Test P<0.001 Z=-4.413
Cues to action before the intervention	1.90±0.95	1.88±0.94	Mann Whitney Test P=0.823 Z=-0.224
Cues to action after the intervention	3.72±1.08	1.81±0.69	Mann Whitney Test P<0.001 Z=-9.815
Health behaviours before the intervention	54.20±7.53	54.45±7.38	Independent *t* test P=0.821 t=0.226
Health behaviours after the intervention	61.85±7.87	55.49±6.74	Mann Whitney Test P<0.001 Z=-6.678

## Discussion

In the present study, the perceived susceptibility score was found to be significantly higher among the experiment group subjects compared to the control group. If individuals perceive themselves to be susceptible to a condition or problem, we can say that their perceived susceptibility about the problems has been increased;^17^ therefore, women in this study who believe that there is a possibility of getting recurrent UTI, they will be interested in performing preventive health behaviours. This might suggest that the intervention had been useful in increasing the perceived susceptibility of the experiment group to promote UTI preventive behaviours among pregnant women.^25^ In line with our study, the other interventional studies also indicated that the score of the perceived susceptibility in the experiment group was significantly increased, compared to the control group, after the education based on HBM.^18^^,^^29^ However, some studies showed that the score of the perceived susceptibility of the experiment group had no significant difference compared to the control group after education based on HBM.^19^

The Present study showed a significant increase in the perceived severity score of the experiment group compared to the control group after the intervention. since, Perceived severity is associated with feelings about the complications of contracting or leaving an illness untreated include both medical and social consequences (for example, death, disability, and pain or change of conditions of work, family life and social relations),^14^ it can be said that the participants of this study believe there is a possibility of getting the complications of recurrent UTI, (such as renal failure, sepsis, adhesions and obstruction); therefore, she will be interested in preventive health behaviours. Our founding is similar to other studies regarding to HIV counselling and testing^30^ and also is in line with Deshpande et al  in terms of the influence of variables such as perceived severity to eat healthfully.^31^ The remarkable finding in another study in Iran^19^ is that the perceived severity score in the control group was decreased after the education based on Health Belief Model on beliefs promotion and screening behaviours of breast cancer. However in the present study, the perceived severity score of the control group was increased after the intervention but this increase has not been significant.

The considered barriers in this study included lack of belief in effectiveness of health behaviours on UTI prevention, complications of the antibiotics, lack of interest in wearing loose underwear and pants and embarrassment to follow up after medication. The remarkable finding in this study was that the perceived barriers score of the experiment group had no significant difference compared to the control group. 

Unlike our founding, previous studies showed that after education based on HBM to promote the health behaviour of women, the score of the perceived barrier in the experiment group has been significantly decreased, compared to the control group.^18^^,^^32^^,^^33^

It should be mentioned that the score of the perceived barrier in the experiment group was decreased compared to before the intervention; however, after intervention the difference between the two groups was not significant. This finding might be interpreted in this way that the increase in the perceived susceptibility of the individuals may lead to an increase in their perception about susceptibility to the illness and to a decrease in their attention to the barriers of the health behaviours.

Reduction in treatment costs, possibility of recurrence and complications were considered as perceived benefits in this study. The results of the present study showed a significant increase in the perceived benefits score of the experiment group compared to the control group, after the intervention. Our founding is in line with Pinto et al.^23^ regarding the retention in diabetes-related pharmaceutical care services.

The results are contradictory to those of another study in which researchers used this model for the education of osteoporosis prevention; they did not report a significant difference in the perceived benefits score of calcium supplement between the experiment and control groups. It could be due to disagreement of people in the community about using or not using nutriment supplement and general doubt about their effectiveness.^34^

Cues to action in this study were checked in a framework of 8 yes-no questions. The questions were about internal and external stimulus which could act as cues to action for the individuals in order to conduct health behaviours preventing UTI. One of the cues to action was internal stimulus (history of UTI) and the others were external stimuli (media, educational booklets, books, friends, family, education, etc). The number of positive answers to these questions was few in both groups so that, in spite of maximum score of this part which was 8, the mean score of the cues to action was 1.90 in the experiment group and 1.88 in the control group. This result underlines the urgent need of women with UTI to the educational health programs in the field of UTI.  

The present study showed that the score of the health behaviours was found significantly higher among the experiment group compared to the control group after the intervention. A body of literature supports the importance of health habits and behaviours play an important role in causing UTI women.^12^^,^^28^ But change in beliefs can lead to changes in health behaviour which contribute to improved health status.^17^ This point shows the importance of our study in promoting the health beliefs as a basis of behavioural change.

Our founding is in line with TAGHDISI^25^ indicating that health education leads to health behaviour improvement about UTI among pregnant women. This result is also in the same line with some other recent studies^24^^-^^26^ about the effect of education based on HBM on different health behaviours.

## Conclusion

Based on the findings of this study, we can conclude that education based on Health Belief Model had effects on the perceived susceptibility, perceived severity, perceived benefits, cues to action and health behaviours of the women suffering from UTI, but it had no significant effect on the perceived barriers. Therefore, it is suggested that health education in the field of preventive behaviours for UTI should be accessible for everyone, especially married women with UTI. It should be suggested that, in the future research, the barriers of health behaviours should be recognized and the impact of education based on HBM on prevention of recurrent UTI need to be studied. 
